# Highly ordered mesoporous silica film nanocomposites containing gold nanoparticles for the catalytic reduction of 4-nitrophenol

**DOI:** 10.3762/bjnano.10.135

**Published:** 2019-07-05

**Authors:** Mohamad Azani Jalani, Leny Yuliati, Siew Ling Lee, Hendrik Oktendy Lintang

**Affiliations:** 1 Kolej PERMATA Insan, Universiti Sains Islam Malaysia, Kompleks PERMATA Insan, Bandar Baru Nilai, 71800 Nilai, Negeri Sembilan, Malaysia; 2 Department of Chemistry, Faculty of Science, Universiti Teknologi Malaysia, 81310 UTM Johor Bahru, Johor, Malaysia; 3 Ma Chung Research Center for Photosynthetic Pigments, Universitas Ma Chung, Malang 65151, East Java, Indonesia; 4 Department of Chemistry, Faculty of Science and Technology, Universitas Ma Chung, Malang 65151, East Java, Indonesia; 5 Centre for Sustainable Nanomaterials, Ibnu Sina Institute for Scientific and Industrial Research, Universiti Teknologi Malaysia, 81310 UTM Johor Bahru, Johor, Malaysia

**Keywords:** catalyst, gold nanoparticle, mesoporous silica, nanocomposite, thermal hydrogen reduction, 4-nitrophenol reduction

## Abstract

We report that transparent mesostructured silica/gold nanocomposite materials with an interpore distance of 4.1 nm, as-synthesized from a templated sol–gel synthesis method using discotic trinuclear gold(I) pyrazolate complex, were successfully utilized for the fabrication of thin film mesoporous silica nanocomposites containing gold nanoparticles. The material exhibited a highly ordered hexagonal structure when subjected to a thermal hydrogen reduction treatment at 210 °C. In contrast, when the material was subjected to calcination as a heat treatment from 190 to 450 °C, the thin film nanocomposites showed an intense *d*_100_ X-ray diffraction peak. Moreover, gold nanoparticles inside the thin film nanocomposites were confirmed by the presence of the *d*_111_ diffraction peak at 2θ = 38.2°, a surface plasmon resonance peak between 500–580 nm, and the spherical shape observed in the transmission electron microscope images, as well as the visual change in color from pink to purple. Interestingly, by simply dipping the material into a reaction solution of 4-nitrophenol at room temperature, the highly ordered structure of the as-fabricated silica/gold nanoparticle thin film composite after thermal hydrogen reduction at 210 °C resulted in an improved catalytic activity for the reduction of 4-nitrophenol to 4-aminophenol compared to the material calcined at 250 °C. Such catalytic activity is due to the presence of gold nanoparticles of smaller size in the silicate channels of the highly ordered mesoporous film nanocomposites.

## Introduction

Mesoporous silica nanomaterials with pore size between 2 to 50 nm [1] have been recently applied to the development of biomedical adsorbents [2–4], drug delivery systems [5–7], catalysts [8–10], as well as supports for metal nanoparticles [11–13] due to their large surface area, good thermal stability, high uniformity, and controllable pore size [1]. These nanomaterials with a hexagonal structure were independently discovered using a layered silicate kanemite as a template to form folded sheet materials (FSM)-16 [14] and cetyltrimethylammonium bromide (CTAB) as a cationic surfactant to form the material Mobil composition of matter (MCM)-41 [15]. Considering their low toxicity, their bio-degradable nature, and the fact that they are inexpensive and highly availability, non-ionic surfactants such as Brij [16] and Pluronic [17] block copolymers have also been employed as templates [18] to form as-synthesized mesoporous (mesostructured) silica with higher acidity and thicker pore walls. For the preparation of mesoporous silica, the templates need to be removed from the silicate nanochannels to allow the formation of porous structures [1]. Generally, this can be carried out by a calcination method at high temperature in the presence of air [1,14–17]. However, this process can also lead to structural damage due to the removal of organic components from the template at high temperature [18]. Since the high quality of mesoporous silica nanomaterials can increase the performance for the above-mentioned applications, it is crucial to find a method for template removal.

As a host for metal nanoparticles, mesoporous matrixes have been extensively used for the controlled growth of gold nanoparticles (AuNPs) [11,19]. For mesoporous silica, gold sources derived from gold(III) chloride trihydrate (HAuCl_4_) solution have been used as a precursor for post-synthetic grafting upon mixing with mesoporous silica [20]. Another method is to utilize the co-condensation reaction by the mixing solution of HAuCl_4_ with surfactants and silica sources during the sol–gel synthesis [21]. However, after template removal at 450 °C or above, both approaches suffer from agglomeration, non-homogenous distribution, and low insertion of AuNPs in the silicate nanochannels. On the other hand, hexagonal mesostructured silica nanomaterials with dense filling of organic functional groups were successfully reported using a functional organic amphiphile surfactant as a template [22–24]. Lintang et al. [25] reported the fabrication of hexagonal mesoporous silica nanocomposites ([Au_3_Pz_3_]_C10TEG_/silica_hex_) using columnar assembly of an amphiphilic trinuclear gold(I) pyrazolate complex ([Au_3_Pz_3_]_C10TEG_) as a self-assembled template. Indeed, the resulting mesostructured silica nanocomposites not only exhibited perfect self-repairing properties, but also worked as a metal ion sensor [26] with excellent phosphorescent sensing and temperature imaging capabilities [27]. Hence, it is very interesting to utilize this nanocomposite for the growth of AuNPs in the silicate nanochannels.

**Figure 1:**
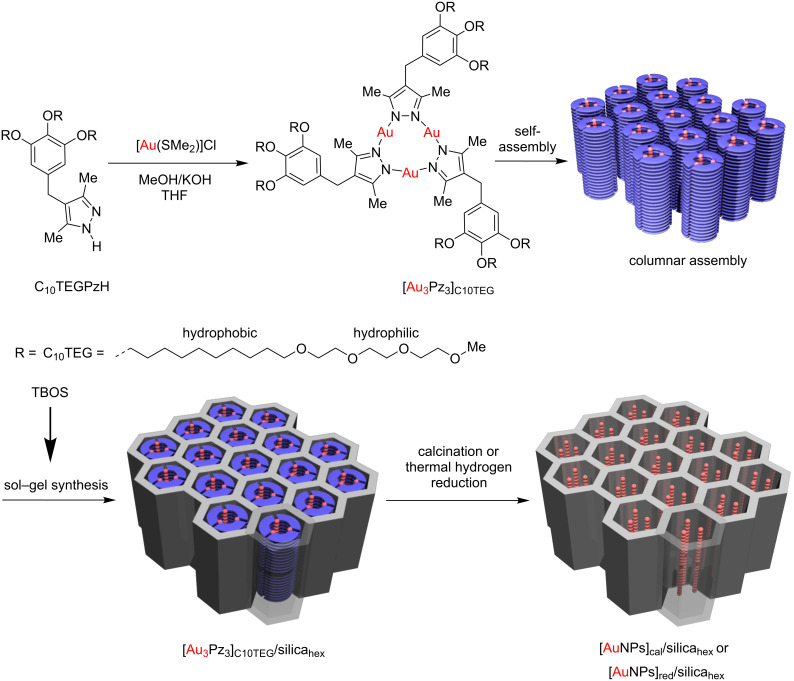
Schematic for the synthesis of [Au_3_Pz_3_]_C10TEG_ from C_10_TEGPzH and the fabrication of [Au_3_Pz_3_]_C10TEG_/silica_hex_ through a sol–gel synthesis with tetrabutyl orthosilicate (TBOS) as a silica source, followed by the synthesis of [AuNPs]_cal_/silica_hex_ or [AuNPs]_red_/silica_hex_ by calcination or thermal hydrogen reduction. The synthetic scheme is adapted from [25].

Recently, mesoporous silica nanomaterials were not only used as a support to control the size of the AuNPs [28], but also to enhance the thermal stability after heat treatment at high temperature [29]. In our previous works, high purity [Au_3_Pz_3_]_C10TEG_ [30] was used to fabricate [Au_3_Pz_3_]_C10TEG_/silica_hex_ for synthesizing AuNPs in the nanocomposites via calcination ([AuNPs]_cal_/silica_hex_) [31] and thermal hydrogen reduction methods ([AuNPs]_red_/silica_hex_) [32]. However, the resulting quality of the mesoporous silica nanomaterials was quite low based on their characteristic diffraction peaks and microscopy images. Therefore, by varying the reduction temperature close to the thermal decomposition of its organic components during calcination and thermal hydrogen reduction methods, we report herein the successful fabrication of highly ordered mesoporous silica film nanocomposites consisting of AuNPs ([AuNPs]_red_/silica_hex_) as shown in Figure 1. Since 4-nitrophenol (4-NP) has been reported as a chemical harmful to human beings due to their highly toxic nature and yet it can be easily found in industrial wastewaters due to its high solubility properties [33], it is very crucial to find an effective method for the degradation and transformation of 4-NP. Generally, the reduction of 4-NP by heterogeneous catalysts in powdered form usually involves a tedious and time-consuming recovery process such as filtration and centrifugation in order to retrieve the catalysts. Hence, we highlight the utilization of thin film nanocomposites [AuNPs]_red_/silica_hex_ as a heterogeneous catalyst for the reduction of 4-NP to 4-aminophenol (4-AP), where a thin film was simply dipped into the reaction system containing an excess of sodium borohydride (NaBH_4_).

## Results and Discussion

### Thermal decomposition of [Au_3_Pz_3_]_C10TEG_

**Figure 2:**
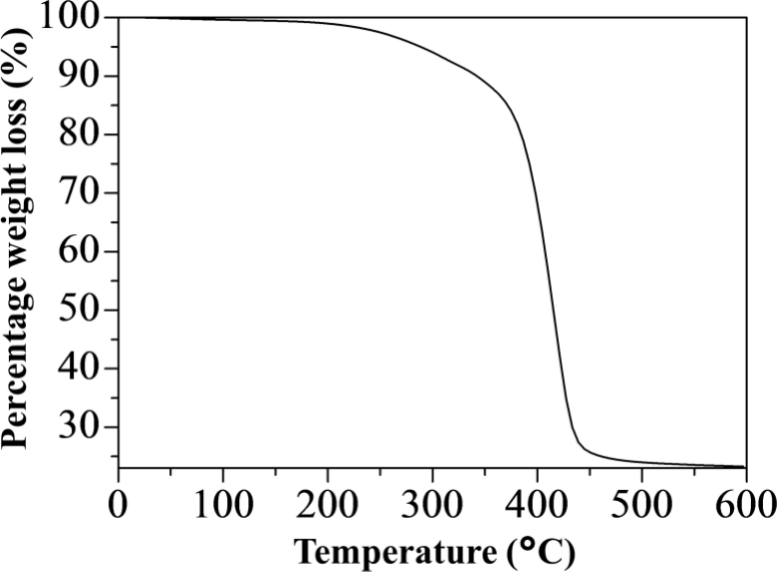
TGA thermogram of [Au_3_Pz_3_]_C10TEG_.

Thermogravimetric analysis (TGA) was used to examine the process of weight loss as a function of temperature change [34]. The thermal behavior of the [Au_3_Pz_3_]_C10TEG_ was further supported by its TGA thermogram as shown in Figure 2. Based on the thermogram, the first weight loss step occurred around 50 to 190 °C at 1 wt %, indicating the removal of physically adsorbed water molecules. The second step took place from 190 to 260 °C due to the chain opening of organic complexes in [Au_3_Pz_3_]_C10TEG_ with weight loss of around 4 wt %. This was followed by the decomposition of long aliphatic alkyl chains and aromatics rings from 260 to 450 °C with 70 wt % loss. The total weight loss was calculated to be around 75 wt %. The remaining weight, at around 25 wt %, can be attributed to the presence of possible residual carbonaceous species [35] and inorganic components [36].

### Structural analysis of the nanocomposites before thermal treatment

**Figure 3:**
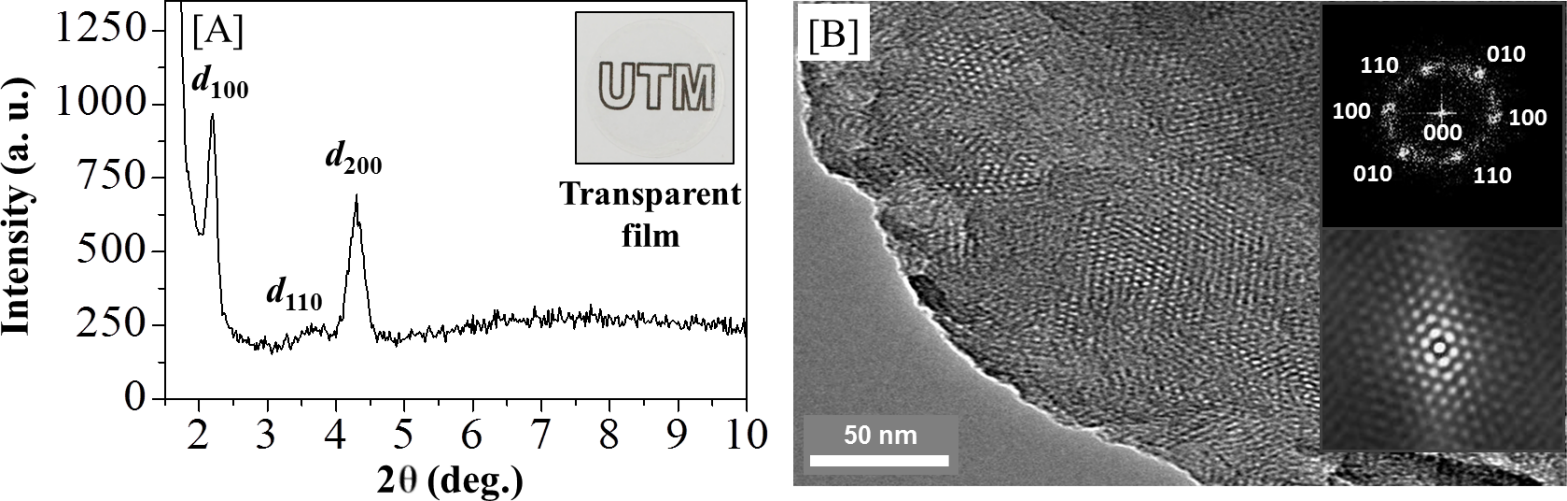
a) XRD diffractogram; the inset is a photograph of the material. b) TEM image; the inset shows the FFT (top) and auto-correlation images (bottom) of [Au_3_Pz_3_]_C10TEG_/silica_hex_.

Generally, the study of the formation of mesostructured silica nanocomposites can be characterized by using X-ray diffraction (XRD) at the small-angle region [37]. Figure 3a shows diffractograms of the transparent mesostructured silica film [Au_3_Pz_3_]_C10TEG_/silica_hex_ (inset figure) having three diffraction peaks at 2θ of 2.2°, 3.7° and 4.3° for *d*_100_, *d*_110_ and *d*_200_, respectively. Such diffraction peaks for [Au_3_Pz_3_]_C10TEG_/silica_hex_ indicate the characteristic hexagonal structure, templated by the discotic liquid crystal of [Au_3_Pz_3_]_C10TEG_ [25], with orientation parallel to the substrate [38]. By using Bragg’s law, the interpore distance of the hexagonal structure at 2θ = 2.2° was found to be 4.1 nm, which is close to the calculated molecular size of the complex with the assumption of a 1 nm wall thickness.

Figure 3b provides transmission electron microscopy (TEM) images of [Au_3_Pz_3_]_C10TEG_/silica_hex_, showing a uniform structure with a hexagonal arrangement. The interpore distance based on this TEM image was calculated to be around 4.0 to 5.0 nm, which was in good agreement with the XRD data. The fast Fourier transform (FFT) and auto-correlation images are shown as inset figures (top and bottom, respectively) in Figure 3b. These reveal the corresponding planes of mesostructured silica with a hexagonal structure [39]. Hence, the structural analysis from XRD and TEM measurements confirmed the formation of a hexagonal structure in the silicate nanochannels of [Au_3_Pz_3_]_C10TEG_/silica_hex_.

### Structural analysis of the nanocomposites after heat treatment

**Figure 4:**
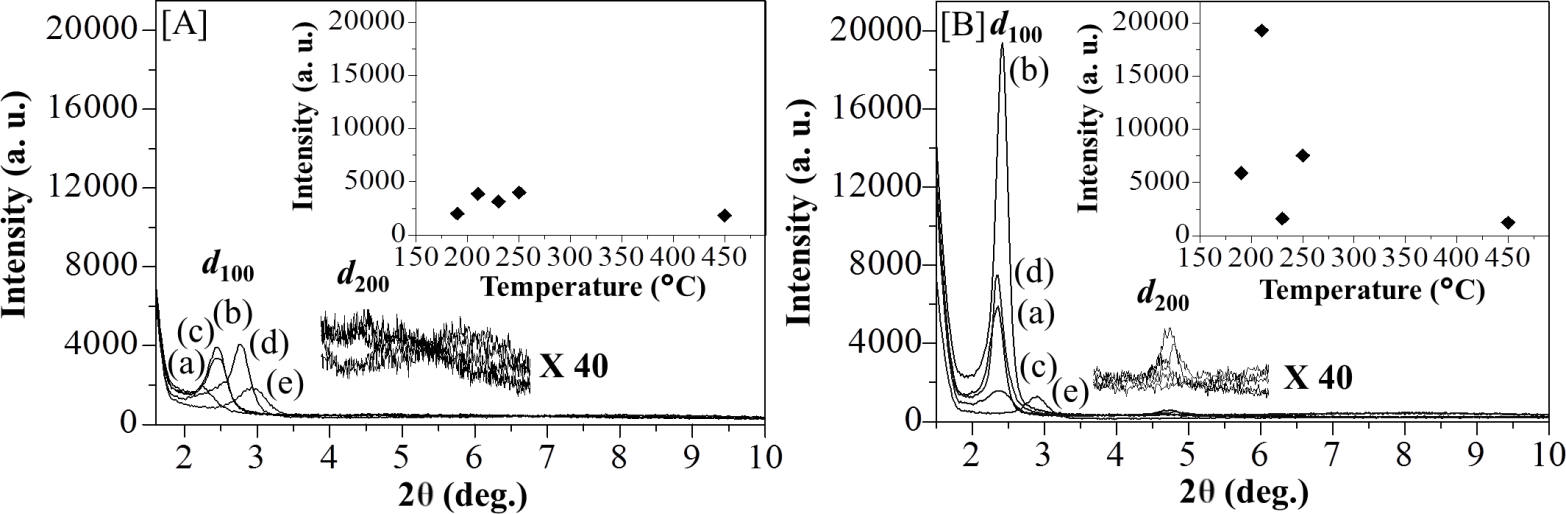
XRD patterns in the small-angle region of a) [AuNPs]_cal_/silica_hex_ films after calcination and b) [AuNPs]_red_/silica_hex_ films after the thermal hydrogen reduction at (a) 190, (b) 210, (c) 230, (d) 250 and (e) 450 °C, where the inset graphs shown the XRD peaks of the maximum intensity of *d*_100_ versus temperature.

The quality of the hexagonal mesostructured silica can be generally evaluated from its ordered or disordered structures by heating at high temperature. Figure 4a,b shows the XRD patterns of [Au_3_Pz_3_]_C10TEG_/silica_hex_ in the range of 190 to 450 °C (plots (a)–(e)). In both methods, the diffraction peaks for *d*_100_ were still preserved due to good thermal stability and the well-ordered pore structure of [Au_3_Pz_3_]_C10TEG_/silica_hex_. Interestingly, by using thermal hydrogen reduction, such preservation of *d*_100_ in the intensity can also be observed even at a temperature as low as 190 °C (Figure 4b, plot (a)). Moreover, the position of the *d*_100_ diffraction peak was shifted to a higher angle with increasing calcination and reduction temperature (Table 1), indicating a small decrease of pore size and unit cell. For example, when the calcination temperature was changed from 190 to 250 °C in increments of 20 °C (Figure 4a, plots (a)–(d)), the position of the *d*_100_ diffraction peaks for the resulting mesoporous silica ([AuNPs]_cal_/silica_hex_) were shifted to a higher angle from 2θ = 2.2° (entry 1, Figure 4a, plot (a)) to 2θ =2.5° at 210 °C (entry 2, Figure 4a, plot (b)) and 230 °C (entry 3, Figure 4a, plot (c)) as well as 2θ = 2.8° at 250 °C (entry 4, Figure 4a, plot (d)). These shifts were obviously observed due to calcining at 450 °C to give the *d*_100_ diffraction peaks at 2θ = 2.9° (entry 5, Figure 4a, plot (e)). Calculations of the interpore distance of the hexagonal structure showed a decrease from 3.9 nm to 3.6 nm at 210 °C (entry 2) and 230 °C (entry 3) as well as a decrease from 3.2 nm and 3.0 nm at 250 °C (entry 4) and 450 °C (entry 5) with a maximum reduction of 0.9 nm due to the shrinkage of the silica walls. Such shifts were also observed when thermal hydrogen reduction was performed at the same temperature (Figure 4b; 2θ = 2.3°, *d* = 3.8 nm) at 190 °C (entry 6, Figure 4b(a)) to 2.4° (*d* = 3.7 nm) at 210 °C (entry 7, Figure 4b(b)), 2.4° (*d* = 3.7 nm) at 230 °C (entry 8, Figure 4b(c)), 2.3° (*d* = 3.8 nm) at 250 °C (entry 9, Figure 4b(d)) and 2.9° (*d* = 3.1 nm) at 450 °C (entry 10, Figure 4b(e)). The decrease in the interpore distance due to the thermal hydrogen reduction method at 450 °C was calculated to be 0.7 nm, which is less than for the calcination method (0.9 nm). Hence, the thermal hydrogen reduction was determined to be the best heat treatment method for the preservation of the hexagonal structure and to limit the disruption and shrinkage of triethylene glycol (TEG) interpenetration into the silica wall when the temperature was increased near the full decomposition of the [Au_3_Pz_3_]_C10TEG_ template.

**Table 1:** Summary of the *d*_100_ XRD peaks, *d*-spacing of mesoporous silica, the crystallite size based on calculations using Scherrer’s equation, and the surface plasmon resonance (SPR) peak maxima of the AuNPs after both types of heat treatments.

	Heat treatment	*d*_100_ XRD peak (deg.)	*d*-spacing of *d*_100_ (nm)	Crystallite size (nm)	SPR band (nm)

1	calcination, 190 °C	2.2	3.9	23	556
2	calcination, 210 °C	2.5	3.6	24	546
3	calcination, 230 °C	2.5	3.6	24	555
4	calcination, 250 °C	2.8	3.2	25	541
5	calcination, 450 °C	2.9	3.0	27	538
6	thermal H_2_, 190 °C	2.3	3.8	18	561
7	thermal H_2_, 210 °C	2.4	3.7	17	563
8	thermal H_2_, 230 °C	2.3	3.8	18	554
9	thermal H_2_, 250 °C	2.3	3.8	19	559
10	thermal H_2_, 450 °C	2.9	3.1	20	550

The quality of the mesoporous silica nanocomposites can be indirectly identified using the intensity of the *d*_100_ diffraction peaks. For samples treated with the calcination method, Figure 4a shows no significant change in the *d*_100_ intensity of [AuNPs]_cal_/silica_hex_. In contrast, for samples treated with the thermal hydrogen reduction method, [AuNPs]_red_/silica_hex_ presented a significantly improved *d*_100_ intensity, even at 190 °C (Figure 4b). When the changes in intensity were compared as a function of temperature (insets in Figure 4a and Figure 4b), it can be observed that the thermal hydrogen reduction at 210 °C provided the best quality of mesoporous silica nanocomposites with a *d*_100_ intensity four times higher than the highest *d*_100_ peak after calcination at 250 °C. By holding and carrying out thermal hydrogen reduction at 210 °C, the composite will start to decompose its organic components in [Au_3_Pz_3_]_C10TEG_ and then form porous structures in mesoporous silica with high quality. It should be noted that [AuNPs]_red_/silica_hex_ at 230 °C showed a significant decrease in the ordered structure, suggesting the competition of a reduction process and the decomposition of organic components. Of interest is the thermal hydrogen reduction at 250 °C which gave even better quality silica film composites compared to the best results using the calcination method.

**Figure 5:**
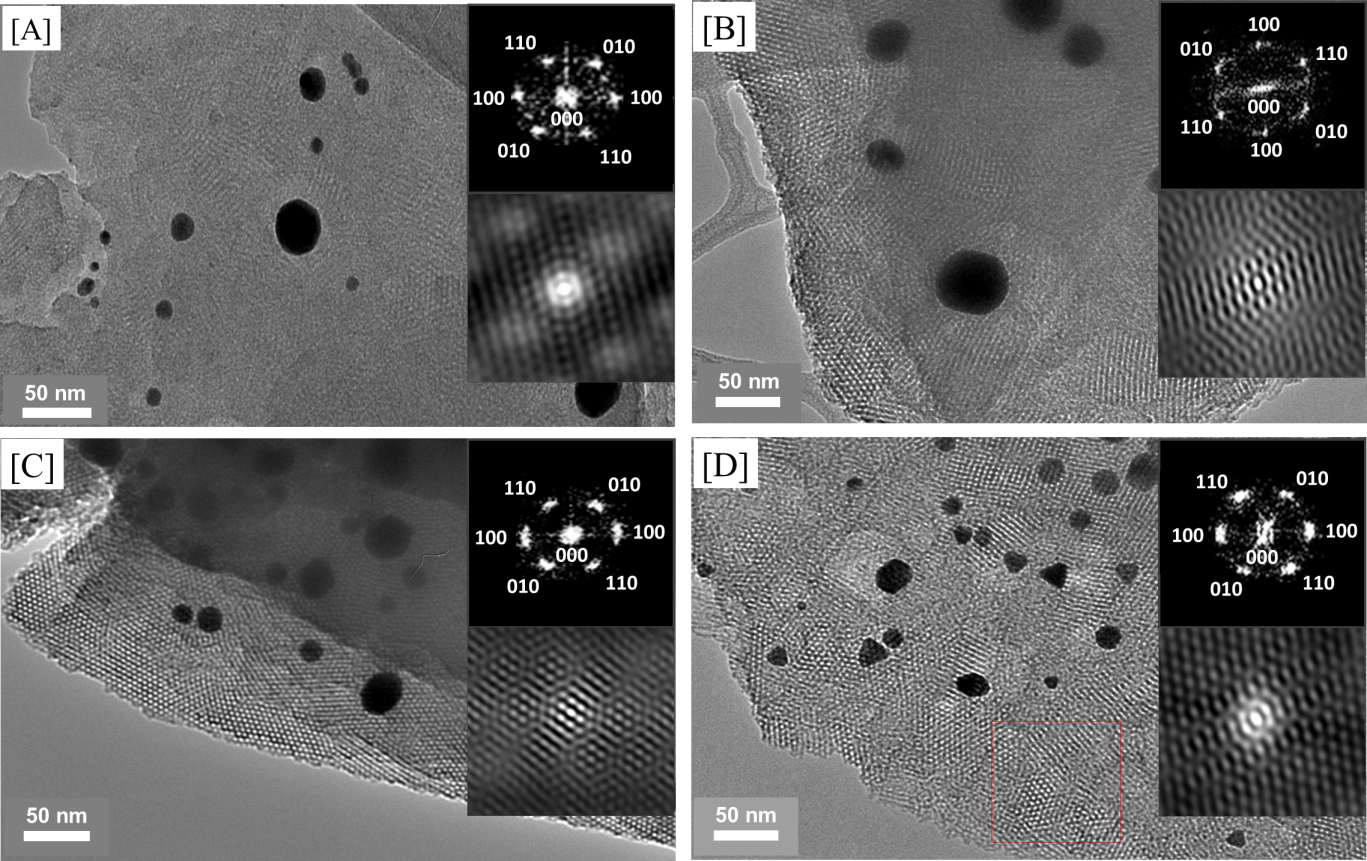
TEM images of a) [AuNPs]_cal_/silica_hex_ films at 250 and b) 450 °C and as well as c) [AuNPs]_red_/silica_hex_ films at 210 and d) 250 °C. The insets show the corresponding FFT (top) and auto-correlation images (bottom).

In our previous reports [31–32], we highlighted the morphology of mesoporous silica composites after both types of heat treatments at 450 and 250 °C by calcination for 3 hours and thermal hydrogen reduction for 2 hours. In this current work, we have only selected the best two samples from each heat treatment process (calcination at 250 °C and thermal hydrogen reduction at 210 °C) for the TEM measurements. Figure 5a and Figure 5b show TEM images of [AuNPs]_cal_/silica_hex_ at 250 and 450 °C, while Figure 5c and Figure 5d show TEM images of [AuNPs]_red_/silica_hex_ at 210 and 250 °C. Interestingly, thin silica [AuNPs]_red_/silica_hex_ at 210 °C (Figure 5c) displayed the best visualization of hexagonal arrangements. This finding was strongly supported by the FFT results which showed intense electron diffraction (top inset figure) and clearly displayed auto-correlation images (bottom inset figure) for the hexagonal honeycomb structure [39]. The high quality of hexagonal arrangement for [AuNPs]_red_/silica_hex_ at 210 °C was supported by its intense diffraction peak of *d*_100_ (Figure 4b(b)).

### Crystallite size analysis of AuNPs

**Figure 6:**
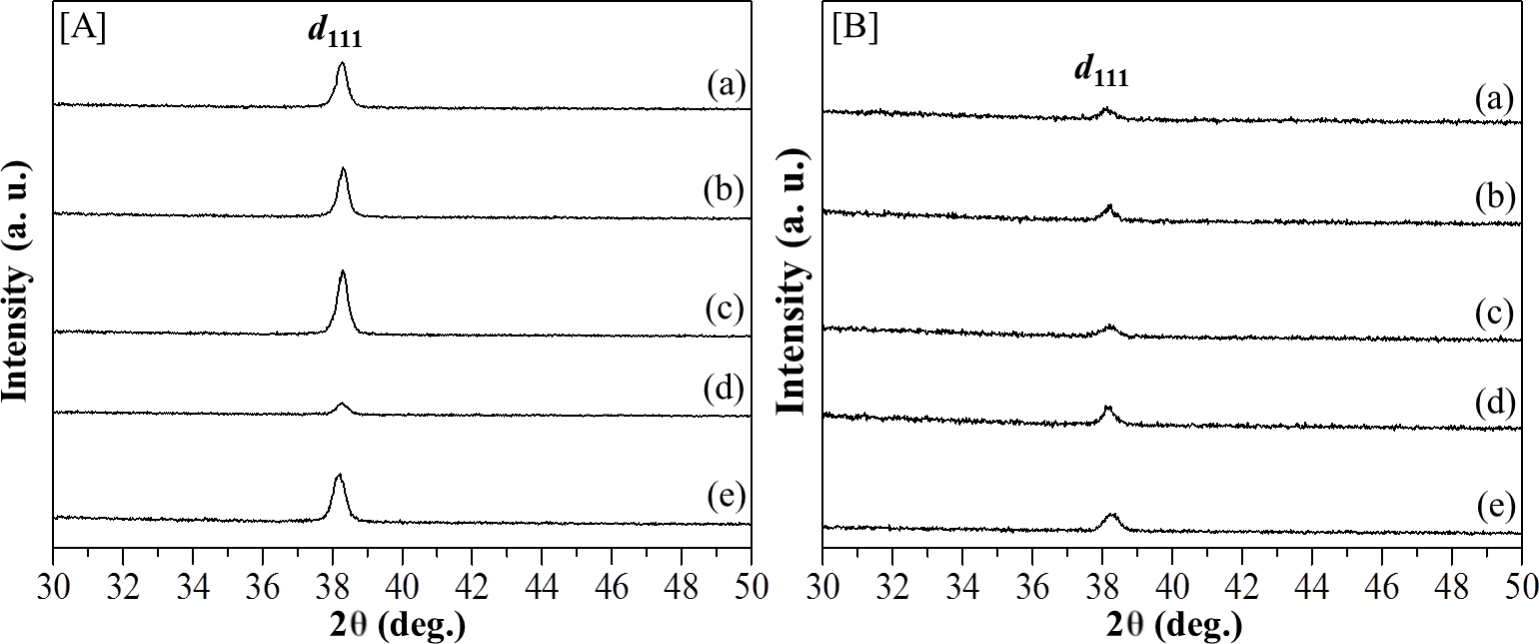
XRD patterns for the wide-angle region of a) [AuNPs]_cal_/silica_hex_ and b) [AuNPs]_red_/silica_hex_ at (a) 190, (b) 210, (c) 230, (d) 250 and (e) 450 °C.

**Figure 7:**
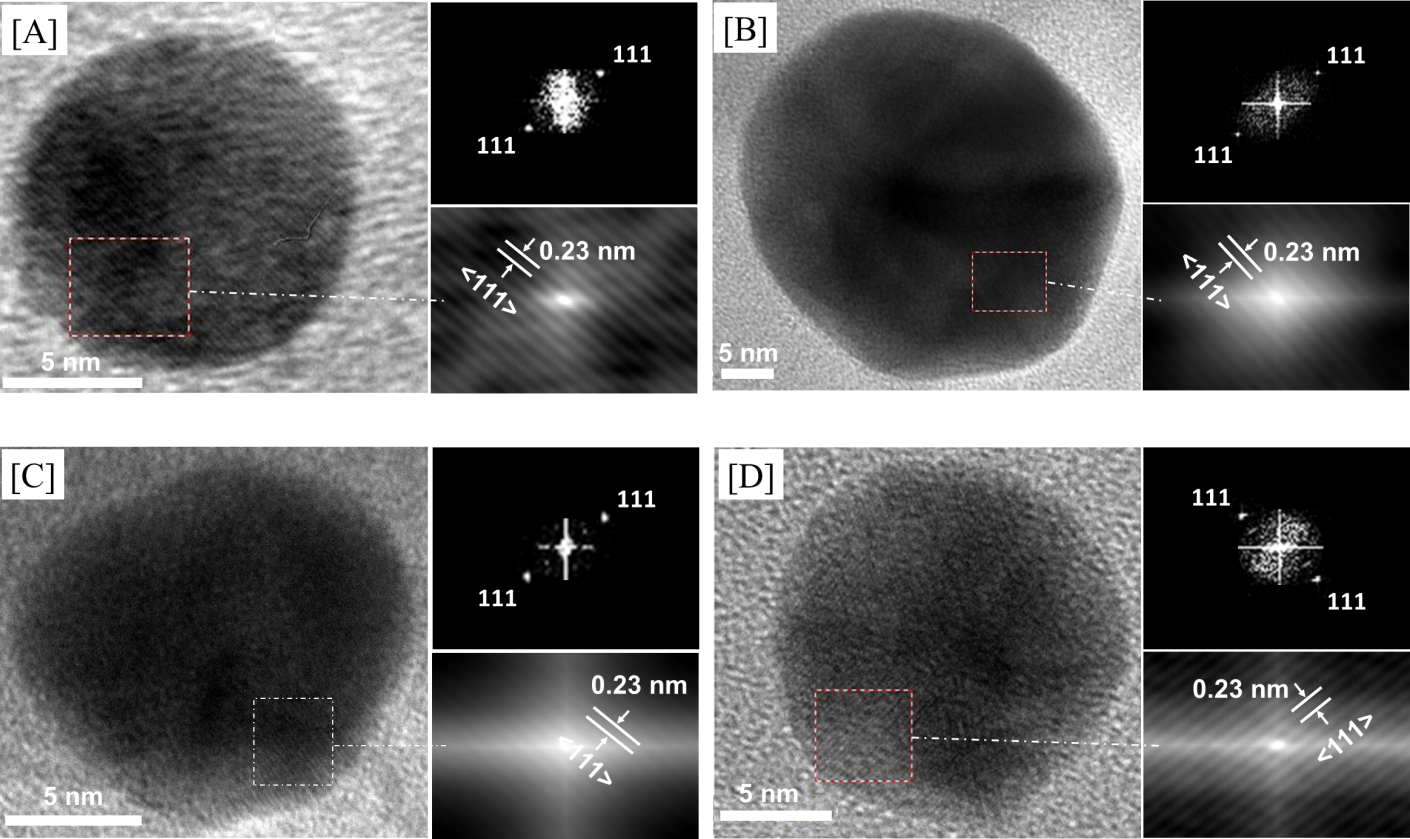
a) TEM images of [AuNPs]_cal_/silica_hex_ films at 250 and b) 450 °C as well as c) [AuNPs]_red_/silica_hex_ films at 210 and d) 250 °C. The insets show the corresponding FFT patterns (top) and auto-correlation images (bottom).

Figure 6 shows the formation of AuNPs based on the X-ray diffraction peaks in the wide-angle area. In this case, diffraction peaks at 2θ = 38.2° were observed for all samples for both heat treatments ((a)–(e)). AuNPs with such particular diffraction peaks were generally confirmed to have a *d*_111_ plane with a cubic phase [40]. In order to determine the crystallite size, Scherrer’s equation was applied and the results are summarized as shown in Table 1. According to the TEM images (Figure 5b), samples treated by calcination or thermal hydrogen reduction at 450 °C (Figure 6a(e) and 6b(e)) resulted in larger AuNPs (around 27 and 20 nm) than expected based on calculations. When thermal hydrogen reduction was applied at 210 °C, the crystallite size of the AuNPs was 17 nm while the TEM image in Figure 5c showed particles with the size in that range. TEM images with magnification from 50 to 5 nm for calcination at 250 and 450 °C (Figure 7a and 7b) as well as thermal hydrogen reduction at 210 and 250 °C (Figure 7c and 7d) showed the presence of AuNPs. All film composites showed AuNPs with an indexed reflection in the FFT pattern corresponding to *d*_111_ with a face centered cubic (fcc) structure at 2θ = 38.2° and a fringe spacing of 0.23 nm (ICDD 98-005-0876). Based on Figure 7, we have also observed from the TEM images that the AuNPs cover the external silicate nanochannels, suggesting a weak interaction between AuNPs with the porous silica structure and the possibility of Ostwald ripening during both heat treatments. Another reason for such an observation could be due to the use of TEM with higher electron energy during the measurement. Moreover, the presence of external AuNPs was suggested due to the sintering effect as reported by others [29]. As the source of the AuNPs, the organic functional groups are arranged inside the silica channels using a template sol–gel synthesis of mesoporous silica with a functional surfactant [22–25]. Due to the heat treatment, the organic functional groups produce AuNPs inside the silicate nanochannels, which may also be found outside the silica surface. Therefore, we strongly believe that the AuNPs in the film composites will be mostly arranged in the silica framework where they can be additionally supported by the TEM 3D tomography at low accelerating voltage with topography-based reconstruction to show the pore orientation at the various angles with the presence of AuNPs (see Supporting Information File 1 for the movie).

### Optical properties of AuNPs

**Figure 8:**
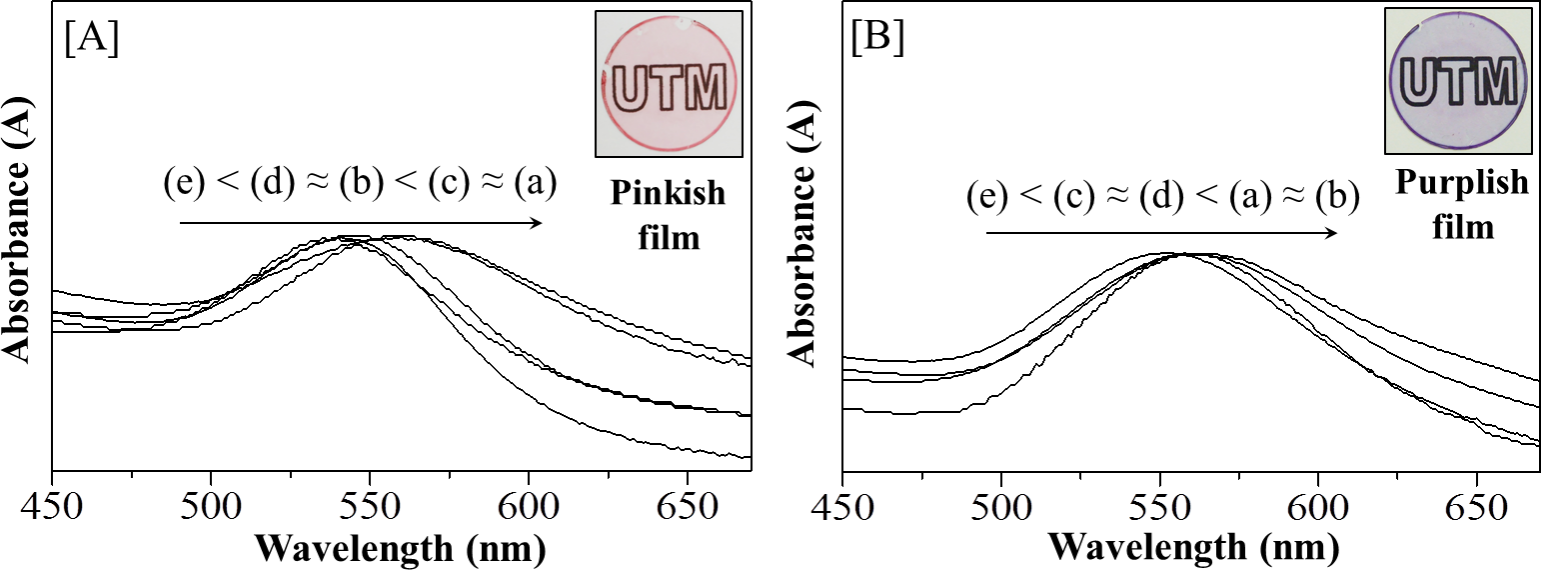
Absorption spectra of a) [AuNPs]_cal_/silica_hex_ and b) [AuNPs]_red_/silica_hex_ films at (a) 190, (b) 210, (c) 230, (d) 250 and (e) 450 °C. The insets show representative photography images for the as-synthesized films after both heat treatments.

Surface plasmon resonance (SPR) peaks in the UV–vis spectrum in the range of 500 to 600 nm can be used to identify the presence of AuNPs [40]. Before calcination or thermal hydrogen reduction was conducted, [Au_3_Pz_3_]_C10TEG_ and [Au_3_Pz_3_]_C10TEG_/silica_hex_ showed absorption bands for π–π stacking of the benzene rings at less than 350 nm without SPR peaks at 500–600 nm [31–32]. After both heat treatments, Figure 8a and 8b shows the SPR peaks at various temperatures and the results are summarized in Table 1. Generally, a red shift of the SPR peaks was observed for both heat treatments from 450 to 190 °C due to the decrease in the average size of the AuNPs [40]. For example, at temperatures as high as 450 °C for both the calcination and thermal hydrogen reduction heat treatment methods (Figure 8a(e) and 8b(e)), the resulting silica film composites gave the lowest SPR bands centered at 538 and 550 nm due to the formation of the largest AuNPs crystallites (27 and 20 nm) as shown in Table 1 (calculated from their XRD diffractograms at wide-angle area as shown in Figure 6a(e) and 6b(e)). Nevertheless, after calcination at 450 °C, the images illuminated by daylight showed a change in color of thin silica film composites from colorless transparent for [Au_3_Pz_3_]_C10TEG_/silica_hex_ to a pinkish color for [AuNPs]_cal_/silica_hex_ (Figure 8a, insert). By using thermal hydrogen reduction at the same temperature, colorless transparent of [Au_3_Pz_3_]_C10TEG_/silica_hex_ was changed to purplish colour of [AuNPs]_red_/silica_hex_ (Figure 8b, insert). Both color changes indicate that different sizes of AuNPs were possibly formed in their thin film composites.

### Catalytic activity

**Figure 9:**
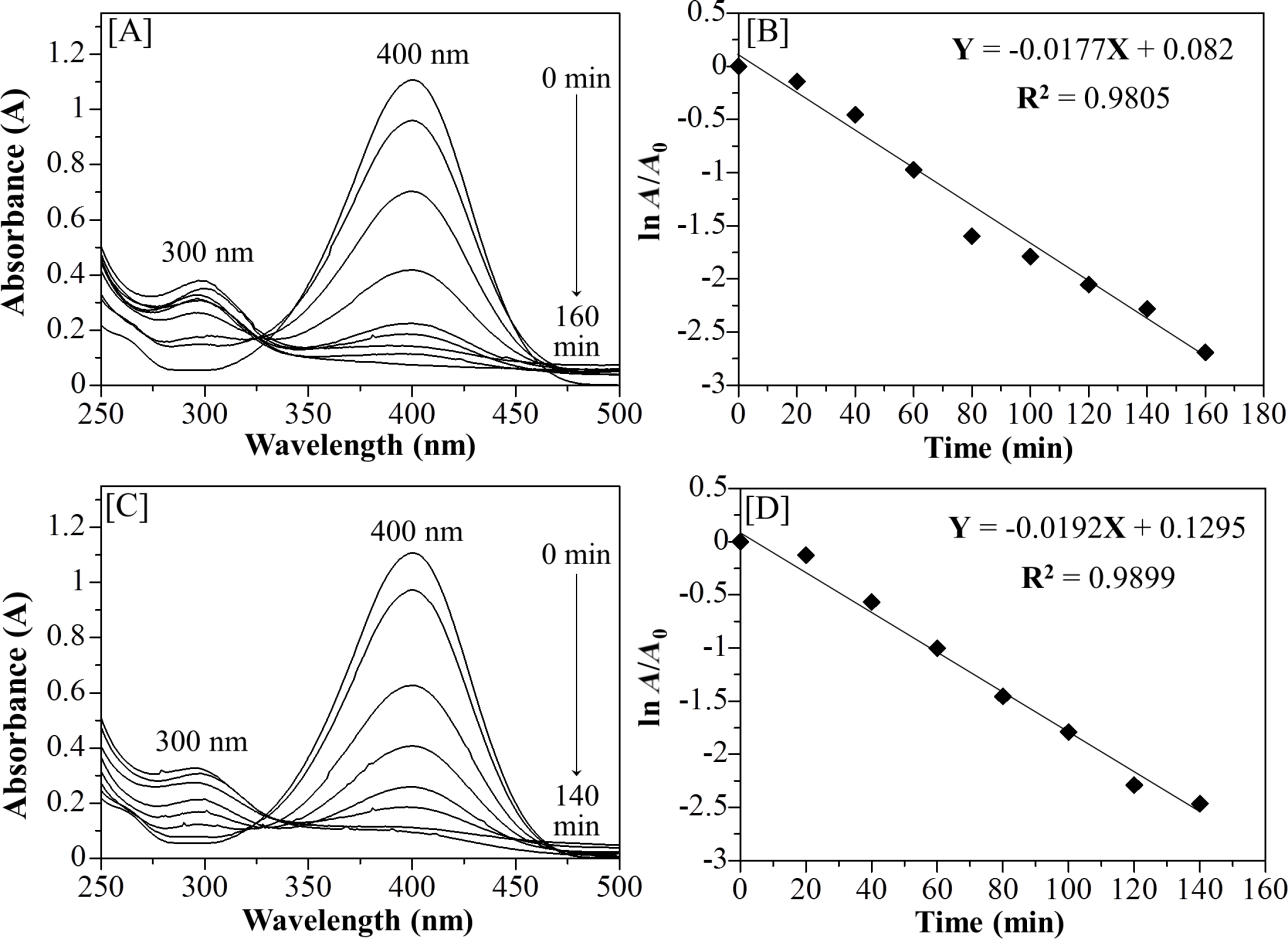
UV–vis absorption spectral changes for the reduction of 4-NP at 20 min intervals over a) [AuNPs]_cal_/silica_hex_ film at 250 °C with b) a corresponding graph of ln *A*/*A*_0_ versus time within 160 minutes. c) The [AuNPs]_red_/silica_hex_ film at 210 °C with d) a corresponding graph of ln *A*/*A*_0_ versus time within 140 minutes.

For the catalysis reaction, only the thin film composites [AuNPs]_cal_/silica_hex_ treated at 250 °C for 3 hours and [AuNPs]_red_/silica_hex_ materials treated at 210 °C for 2 hours were selected because these thin films displayed the best quality within the respective heating methods. In the preparation of the 4-NP solution, the addition of NaBH_4_ in excess caused a red shift of the absorption spectrum from 315 nm to an intense peak at 400 nm due to the presence of 4-nitrophenolate ions [41]. By simply dipping the thin film sample into a 4-NP solution, the reaction was determined to be complete based on the time taken for the peak at 400 nm of 4-NP to completely disappear, while formation of a new peak at 300 nm, corresponding to the formation of 4-AP, was detected and then enhanced. In addition, a color change of the 4-AP solution from yellowish to colorless was also observed at the end of the catalytic reaction. After the calcination of the thin film at 250 °C, the catalytic reduction was completely observed within 160 minutes as shown in Figure 9a. The rate constant (*k*) for this reaction was calculated to be 1.77 × 10^−2^ min^−1^ based on the slope in the graph of ln *A*/*A*_0_ versus time in Figure 9b. Interestingly, the thin film [AuNPs]_red_/silica_hex_ at 210 °C showed an improved catalytic activity, in which the reaction was completed faster – within 140 minutes as shown in Figure 9c with *k* = 1.92 × 10^−2^ min^−1^ (Figure 9d). Since AuNPs were the active site in this reaction, the increase in the catalytic activity of the film composite [AuNPs]_red_/silica_hex_ at 210 °C is strongly suggested by the small particle size of the as-synthesized AuNPs in high-quality mesostructured silica. Since the NaBH_4_ concentration largely exceeded that of 4-NP (and remains constant throughout the experiment), both films were found to follow pseudo-first order kinetics [41]. This reduction followed the Langmuir–Hinshelwood model, where both reactants (4-NP and BH_4_^−^) were adsorbed on the AuNP surface at a fast rate. In the next step, electron transfer from the hydride ion to the AuNPs occurred to give the hydrogen atom that later will react with 4-NP. Finally, 4-AP was formed and dissociated from the AuNP surface [42]. The presence of an isobestic point in Figure 9a,c further proved that only one product, 4-AP, was formed [43–44]. Compared to other reports, the AuNP–film catalyst in this work showed higher catalytic activity for the reduction of 4-NP to 4-AP which was 10 times (0.199 × 10^−2^ min^−1^) and 4 times (0.45 × 10^−2^ min^−1^) higher than for AuNPs prepared from *Gnida glauca* leaf and stem extracts [45]. Another report by Shende et al. [46–47] demonstrated that AuNPs prepared from *Litchi Chinensis* peel extract and *Platanus Orientalis* leaf extract showed 1000 times lower catalytic activity with rate constants of 0.00136 × 10^−2^ and 0.00191 × 10^−2^ min^−1^. Hence, the AuNP–film catalyst in the nanocomposite of this work shows the importance of mesoporous silica as a framework for growing AuNPs in the silicate nanochannels with higher ordered nanostructure. In particular, the amount of AuNPs in our AuNP–film catalyst was only 0.04 mg, indicating that it is a good catalyst even with such a small amount and with a simple method for the catalytic reduction of 4-NP to 4-AP. The turnover frequency (TOF) value of [AuNPs]_red_/silica_hex_ at 210 °C as a thin-film catalyst was calculated to be 0.02 min^−1^, which is higher than that of [AuNPs]_cal_/silica_hex_ at 250 °C with a value of 0.01 min^−1^. Such catalytic activity of the AuNP–film catalyst prepared by thermal hydrogen reduction at 210 °C is due to its higher TOF value [48]. Moreover, in the same catalytic reduction of 4-NP, this TOF value is comparable to Au-Fe_3_O_4_ as a bifunctional catalyst [49]. The catalyst is active to reduce all 4-NP to 4-AP, giving a linear decomposition rate within the reaction time. Unfortunately, we found that the AuNP–film catalyst was pulled out of the glass substrate. Therefore, reusability tests were very difficult [50–51] since we only used a very small amount of the catalyst. We are now in the process of developing a new method to construct a strong coating of the AuNPs on the substrate.

## Conclusion

By using calcination and thermal hydrogen reduction in the range of 190 to 450 °C, we have demonstrated that mesoporous silica/gold nanoparticle thin film composites with a hexagonal structure could be successfully fabricated from as-synthesized mesostructured template materials. The best quality of the silica/gold film nanocomposites was found by thermal hydrogen reduction at 210 °C as determined from its intense *d*_100_ peak and clear TEM image with a hexagonal alignment of the nanopores. Both heat treatment methods could be successfully used to produce gold nanoparticles in the silica film nanocomposites based on the results of diffractograms showing *d*_111_ peaks at 2θ = 38.2°, TEM images with spherical particles and a fringe spacing of 0.23 nm, and the presence of SPR bands in the range between 500 to 600 nm, as well as the photography images at daylight showing a change in color from purplish-pink to dark purple. By simply dipping the material into a 4-NP solution, both nanocomposite films, [AuNPs]_cal_/silica_hex_ (after calcination at 250 °C) and [AuNPs]_red_/silica_hex_ (after thermal hydrogen reduction at 210 °C), were able to act as a heterogeneous catalyst for the reduction to 4-AP. Indeed, the [AuNPs]_red_/silica_hex_ film was found to have a higher catalytic activity with a rate constant of 1.92 × 10^−2^ min^−1^ due to the presence of small gold nanoparticles grown within the high quality mesoporous silica structures.

## Experimental

### Instrumentation

X-ray diffraction (XRD) measurements were carried out at on a Bruker D8 Advance diffractometer with Cu Kα radiation. Ultraviolet–visible (UV–vis) spectra were recorded on a Thermo Scientific model GENESYS 10S UV–vis spectroscopy. Transmission electron microscopy (TEM) images were obtained by using a JEOL JEM-2100 device operating at 200 kV. For the TEM tomography, the sample was visualized using a Hitachi HT7700 instrument for high-resolution imaging at low accelerating voltage (80 kV) where the 3D reconstruction was performed using a Hitachi EMIP tomography acquisition. Thermogravimetric analysis (TGA) was performed using a Mettler-Toledo TGA/SDTA851e device at a heating rate of 10 °C min^−1^. For the fabrication, the sol–gel solution was spin-coated on a quartz plate using a Laurell spin coater, model WS-400-6NPP-LITE. Calcination was carried out under atmospheric conditions using a Nabertherm model LE6/11 muffle furnace, while the thermal hydrogen reduction was carried out under hydrogen gas using a Carbolite (model STF 15/610) tube furnace. Photographic images were captured using a Panasonic model DMC-FZ38 digital camera. Elemental analysis was performed using inductively coupled plasma optical emission spectroscopy (ICP-OES) on an Agilent model 700 series spectrometer where the samples were digested in 5 wt % aqua regia. The catalytic performance was studied via a Shimadzu DR UV–vis spectrophotometer (UV-2600) under liquid set-up with the fast scanning method using a standard 3 mL quartz cell.

### Fabrication of [Au_3_Pz_3_]_C10TEG_/silica_hex_ using sol–gel synthesis

[Au_3_Pz_3_]_C10TEG_ was firstly prepared as a pale-yellow sticky solid in 69% yield using a synthetic scheme as shown in Figure 1. The fabrication of mesostructured silica/gold thin film nanocomposites ([Au_3_Pz_3_]_C10TEG_/silica_hex_) was carried out using a templated sol–gel synthesis according to our previous synthetic protocol [25] as shown in Figure 1 with mole ratios of [Au_3_Pz_3_]_C10TEG_/TBOS/EtOH/HCl/H_2_O 1:60:504:1.2:266. Subsequently, a medium comprised of dry ethanol (61.6 mg, 1.3 mmol), deionized water (11.9 mg, 0.7 mmol) and hydrochloric acid (0.3 mg, 2.9 × 10^−3^ mmol) was prepared before being added to [Au_3_Pz_3_]_C10TEG_ (10.0 mg, 2.5 × 10^−3^ mmol). The mixture was left to dissolve for 20 minutes before tetrabutoxysilane (TBOS, 48 mg, 1.49 × 10^−1^ mmol) was added into the solution. The sol–gel solution was covered with aluminum foil and aged for 12 hours at room temperature. For fabrication as thin films, 70 µL of the final sol–gel solution was spin-coated on a glass substrate to produce a colorless transparent thin film (around 0.5 mg of composite was successfully coated). It was followed by air-drying for a day at ambient temperature.

### Synthesis of [AuNPs]_cal_/silica_hex_ and [AuNPs]_red_/silica_hex_

Thin film [Au_3_Pz_3_]_C10TEG_/silica_hex_ was placed in ceramic crucibles and calcined using a muffle furnace from 190 to 450 °C for 3 hours at a heating rate of 1 °C min^−1^. Another heat treatment, thermal hydrogen reduction, was performed using a tube furnace by placing the thin film into the center of the tube with the same temperature treatment for 2 hours with flow rate of hydrogen gas in 25 mL min^−1^.

### Catalytic reduction of 4-NP

The catalytic reduction of 4-NP to 4-AP was studied using the resulting thin films [AuNPs]_cal_/silica_hex_ and [AuNPs]_red_/silica_hex_. Typically, the catalytic study was conducted by firstly preparing 4-nitrophenolate ions as a yellow solution from the mixture of the 4-NP solution (5.0 mL, 0.3 µmol) and NaBH_4_ powder (23.0 mg, 600.0 µmol) by stirring for 30 seconds. The thin film catalysts were dipped into 3 mL of 4-nitrophenolate ion solution containing a small magnetic stirrer at 1200 rpm until the absorption peak of 4-NP at 400 nm completely disappeared.

## Supporting Information

File 1 TEM 3D tomography video of the AuNP film nanocomposites.
